# Concealed information revealed by involuntary eye movements on the fringe of awareness in a mock terror experiment

**DOI:** 10.1038/s41598-020-71487-9

**Published:** 2020-09-01

**Authors:** Gal Rosenzweig, Yoram S. Bonneh

**Affiliations:** 1 Faculty of Law, Bar-Ilan University, Ramat Gan, Israel; 2 School of Optometry and Vision Science, Faculty of Life Science, Bar Ilan University, Ramat Gan, Israel

**Keywords:** Consciousness, Perception, Cognitive neuroscience, Oculomotor system, Visual system

## Abstract

Involuntary eye movements during fixation are typically inhibited following stimulus onset (Oculomotor Inhibition, OMI), depending on the stimulus saliency and attention, with an earlier and longer OMI for barely visible familiar faces. However, it is still unclear whether OMI regarding familiarities and perceptual saliencies differ enough to allow a reliable OMI-based concealed information test (CIT). In a “mock terror” experiment with 25 volunteers, 13 made a concealed choice of a “terror-target” (one of eight), associated with 3 probes (face, name, and residence), which they learned watching text and videos, whereas 12 “innocents” pre-learned nothing. All participants then watched ~ 25 min of repeated brief presentations of barely visible (masked) stimuli that included the 8 potential probes, as well as a universally familiar face as a reference, while their eye movements were monitored. We found prolonged and deviant OMI regarding the probes. Incorporated with the individual pattern of responses to the reference, our analysis correctly identified 100% of the terror targets, and was 95% correct in discriminating “terrorists” from “innocents”. Our results provide a “proof of concept” for a novel approach to CIT, based on involuntary oculomotor responses to barely visible stimuli, individually tailored, and with high accuracy and theoretical resistance to countermeasures.

## Introduction

It is now well established that concealed memories can be detected via physiological measures^1^. In a recent study^2^, we reported that involuntary eye movements and their inhibition during passive viewing are sensitive to face familiarity. This suggests a potential method for detecting concealed memories, which we successfully explored in the current study. In the following, we describe involuntary eye movements and the phenomenon of oculomotor inhibition, as well as a summary of the current research and methods on concealed information.

### Involuntary eye movements, perceptual deviance, and familiarity

Our eyes move involuntarily, even during fixation of gaze, in a random-walk-like movement and with occasional small saccades or microsaccades^3,4^. When we are consciously perceiving a stimulus onset, these microsaccades are first inhibited for a short period of time, then disinhibited and their rate increases before returning to baseline (see a review in^5^). This oculomotor inhibition (OMI) phenomenon has been linked to attention shifts, stimulus saliency, and anticipation, which determine its time course^5–16^. The OMI is typically shortened with sensory saliency, such as contrast^8^, but it is prolonged for perceptual oddballs or deviance (surprise)^12,17^ and when making a choice^13^, presumably in relation to the processing time required for the choice.

We have recently found that face familiarity prolongs the OMI, as well as shortens its onset^2^. Importantly, the effects of familiarity on the OMI were obtained in passive viewing, on the fringe of awareness, using very short presentations that were immediately masked, similar to a previous P300 EEG study that used masking by rapid serial presentation (RSVP)^18^.

### The concealed information test and the oddball paradigm assumption

The concealed information test (CIT) is a method developed to reveal authentic memory traces. It was designed to objectively reveal personal knowledge, without a report, to prevent deception, and to bypass an inability to report. During CIT, subjects are exposed to repeated serial stimuli, including both natural stimuli and personally significant items (termed “probes”), while their physiological response is measured and averaged in order to detect oddball effects in response to the probes (CIT Protocol). It is assumed that the probes elicit an orienting response due to their deviant appearance as familiar and significant^19–21^. In addition to orienting, there is evidence of an arousal inhibition effect that is applied by the subjects to conceal their orienting activity, and its measure is used for detecting deception^22^. Typically, CIT measures physiological responses such as heart rate and skin conductance^19^, neural responses, primarily the P300 brain wave^23^, as well as eye movements, eye blinks, and pupil dilation^24–27^. These measures typically require a serial repetitive presentation for averaging; as a result, false positives could occur due to an arbitrary orienting and to the observer’s fatigue as well as biological noise, affecting the signal-to-noise ratio (SNR) as in the P300 BCI methods^28^. This suggests that a possible tradeoff exists between accuracy and susceptibility to deception using CIT methods, as ways to increase the SNR such as longer exposures or more repetitions for averaging, could provide more opportunities for deception (e.g.^29^).

### Involuntary eye movements and the concealed information test

Given the ability to detect familiarity via differences in OMI in passive viewing^2^, we can consider its potential use for CIT. First, the use of masked stimuli on the fringe of awareness^18^ reduces the ability for deception. Second, random saliencies induced by sensory properties (like contrast) will shorten the OMI^8^, increasing the deviance but with an effect opposite to familiarity, which prolongs the OMI, and hence should reduce the random saliency (noise) problem. However, there could also be cases of random perceptual deviance. It is currently unknown whether the OMI in response to familiarity and to a random perceptual deviance are similar, possibly due to a common dependence on attention mechanisms, or alternatively, differ, perhaps due to a distinctive early recognition of familiarity^2,30^. In both cases, the OMI could potentially serve as a tool for a Concealed information test (CIT), which is the goal of the current study.

### The current study

**Figure 1:**
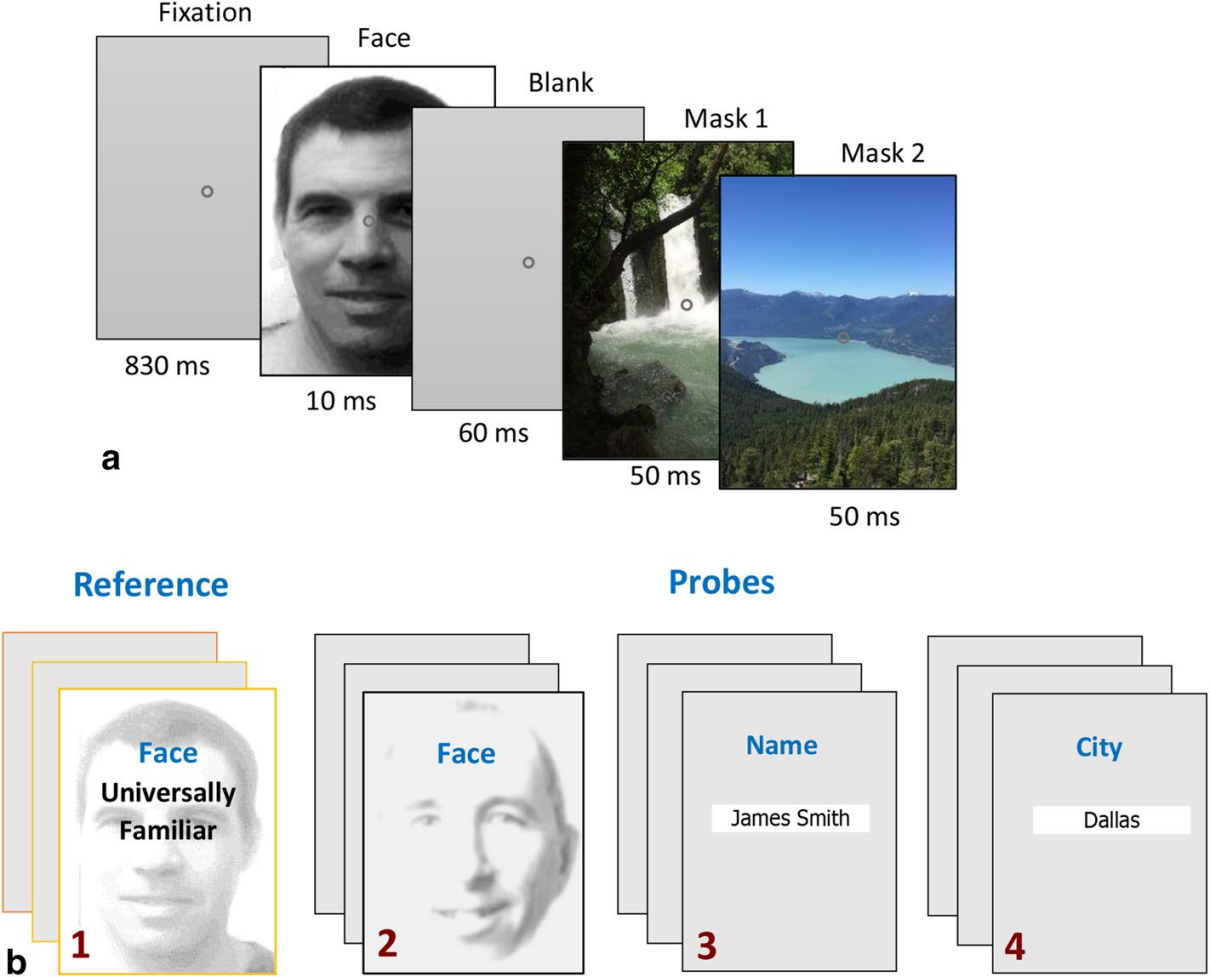
Experimental paradigm. In each trial of the basic paradigm, participants passively viewed a sequence of images as shown (**a**), with one face image in gray levels and two mask color images, with a temporal sequence as shown (left to right, duration specified for each display), making the face barely visible. Trials (96 in a run, 3 runs per experiment) were repeated automatically at 1 Hz rate showing a random sequence of faces (from 8), one familiar. The basic paradigm was used 4 times as depicted in (**b**): (1) a universally familiar face among non-familiar faces, as a Reference, (2–4) 8 suspected mock-terror targets, one of which is a probe for each of the study participants, with (2) faces, (3) family names and (4) city names of the suspected mock-terror target, in black font text on a small white patch. The names in the Figures (3, 4) are for illustration only. The basic paradigm was identical to Rosenzweig and Bonneh^2^.

The aim of the current study was to apply our OMI method for detecting face familiarity as a novel method for a concealed information test in a semi-realistic scenario. For that purpose, we designed a mock terror experiment in which participants of the study group chose a “terror target” associated with 3 probes (face, name, and residence), which they learned using ~ 20 min of video, text, and images (see “Methods” section). All participants were then presented with four slideshows in a passive viewing of briefly flashed images (8 in random order), barely visible due to backward masking, repeated at 1 Hz (Fig. 1, see “Methods” section). These slideshows included one with a universally familiar face among 7 neutral faces, followed by 3 runs with eight potential probes of 3 kinds (face, text name, and residence, see “Methods” section). The results of the high-quality eye tracking data allowed us to identify correctly (100%) all the individually chosen mock terror targets based on oculomotor deviance, as well as to distinguish with high precision between the study group members and an “innocent” control group.

## Methods

### Participants

Twenty-five volunteers with normal or corrected-to-normal vision participated in the experiments. They were divided into a study group (n = 13, 9 males, mean age = 37.9 years, SD = 7.3) and a control group (n = 12, 6 males, mean age = 34.5 years, SD = 5). They were recruited from university students and friends. The project, including the experimental protocol, was approved by the ethics committee (IRB) of Haifa University, and the methods were carried out in accordance with the IRB guidelines and regulations. Informed consent was obtained from all participants.

### Apparatus

The stimuli were displayed on a 22″ CRT monitor, with a refresh rate of 100 Hz and a background luminance of 3.2 cd/m^2^. The resolution was 1,024 × 768 pixels and the display occupied 33.4° × 25.4°. The experiments were conducted in dim light. Due to issues with the CRT availability at the end of the project, five control subjects were recorded with Eizo Foris FG2421 at the same refresh rate (100 Hz) and 1920 × 1,080 resolution, keeping the same stimulus dimensions and brightness. We recorded eye movements with the Eyelink 1000 eye tracker (SR Research, Ontario, Canada) running monocularly (right eye) at a 500 Hz sampling rate, with the head stabilized via a forehead and chin rest. Viewing was binocular from 60 cm. We performed a standard 9-point calibration before each session, although the exact position of the eyes had little importance in the current study. We presented the stimuli using an in-house-developed stimulus presentation platform for psychophysics, eye-tracking, and EEG experiments (PSY) developed by Y.S.B., running on a Windows PC.

### Stimuli and procedures

The participants of the study group (n = 13) took part in a mock terror experiment. They were asked to make a concealed choice of a “terror target” by selecting a number for choosing one of 8 world-wide public male figures unknown to them. A picture of the selected figure, his name, and his residence city were selected as 3 probes for terror target identification. All the probes were South American names and faces to reduce possible early acquaintance, and neither of the observers reported recognizing either of them. After making their choice, which was recorded but kept hidden from the experimenter, they were asked to learn about the chosen target by reviewing for 20 min a set of web links, including watching authentic videos and reading text about the figure, his name, and the city where he resides. Another group of “controls” (n = 12) pre-learned nothing. Following the initial phase (learning by the study group), all participants underwent a series of short (~ 2 min) eye tracking runs, passively watching 4 sets of slide shows of pictures and text repeated 3 times each, making a total of 12 runs over ~ 25 min. The experimental paradigm is described in Fig. 1; the basic stimulus sequence of one epoch is shown in Fig. 1a. It starts with 830 ms of fixation at a central static fixation point (0.128 in diameter) on a gray background (3.2 cd/m^2^), followed by a monochromatic facial or text image (one of 8) flashed for 10 ms, a blank screen (60 ms), and two successive colorful "relaxing" images, 50 ms each, selected at random from a set of 30 images. All face and mask images were 360 × 480 pixels in size. All faces were similar in luminance (with average pixel values in log units of 2.10, SD = 0.08) and RMS contrast (average = 15.8, SD = 2.4). The small differences could not have contributed to the classification of “innocence”, since both groups viewed the same images and could not have contributed to the identification, since every face could have been a target for one subject and a distracter for the other, depending on their prior choice of the target (1–8). The text images (family names, city names) were in black Ariel font on a white rectangular background patch of 180 × 34 pixels and with a luminance of ~ 80 cd/m^2^**,** on the same dark gray background.

This basic sequence was used in 4 different settings, each repeated 3 times (3 ex-experimental runs) as depicted in Fig. 1b: (1) a universally familiar face among 7 non-familiar distracters (as in^2^), (2) Face, (3) Name, and (4) City names, making 3 sets of potential probes. Within a run, each face or text image (among the 8 faces) was presented 12 times in random permutation order, with a total of 96 presentations per run. Participants passively viewed the sequences of stimuli with no former instructions other than fixating on the static central fixation point and paying attention to the presented stimuli.

### Data analysis

The goal of the analysis was to detect the concealed information of familiarity from oculomotor measures, both in terms of identification of the mock terror target for the study group, and for classification of “innocence” vs “involved” by distinguishing the controls from the study group. For that purpose, we compared event-related measures of microsaccades in response to a *familiar face* vs. *unfamiliar faces* and all eight potential probes for the 3 kinds (face, name, and city). These included rate modulation functions and oculomotor RT measures (microsaccades), as used in our previous studies^8,13,31^, as well as new measures of “deviance” for terror target identification and “innocence” classification as described next. Data analysis was carried out using in-house software written in Matlab (The Mathworks, Natick, MA), developed by Y.S.B. The analyses were developed in part post-hoc, i.e. optimized to the obtained experimental results. This weakness is discussed under “Limitations”.

#### Microsaccade and blink detection

Microsaccades were detected using the algorithm introduced by^16^ as implemented in^8,13^. The details are repeated here for completeness. Raw data were first smoothed using local linear regression fitting (the LOWESS method, with a span of 25 ms) to optimize microsaccade extraction. Microsaccades were detected as intervals in which the velocity exceeded a threshold defined as eight median standard deviations of the horizontal and vertical velocities (λ = 8). The minimal microsaccade duration was set to 9 ms. The permitted velocity range was 8°–150°/s and the permitted amplitude range was 0.08°–2°. Eye movements outside these ranges were rejected. The rejection rate varied across participants and was in the range of 0–33%, with an average of 4.1%. When microsaccades were analyzed, periods of missing data, such as during blinks, were locally discarded from further analysis with an additional margin of 100 ms, without discarding the whole epoch.

Eye blinks were detected as in^13^. Although the blinks were informative regarding familiarity, they were less reliable than the microsaccades. We decided not to include them in the current analyses.

#### Total drift calculation

An estimate of the total retinal slip including drift and microsaccades was calculated in order to assess the dependence of the results on accurate microsaccade detection as in^32^. It was calculated for each axis as the range of positions (max minus min) within a sliding window of 0.15 s in steps of 10 ms. Drift estimates greater than 3° were discarded from further analysis. The overall drift was calculated as the square root of the sum of squares (RMS) of the drift in the two axes.

#### Oculomotor modulation functions

The continuous oculomotor data, including microsaccades’ onsets, total drift, and pupil size, were first cut into epochs triggered by stimulus onset, with a time range of − 0.5 s to 1.5 s relative to the trigger (time 0). Epoch data were then used to compute the Oculomotor Modulation Functions (OMF) as averages across epochs. The microsaccade rate modulation function was calculated as in^13^. Rates were computed by convolving a raw rate estimate of one microsaccade (or blink) per sample duration at the time of onset with a causal kernel^33^. The oculomotor modulation functions (for microsaccade rate, total drift, and pupil size) were first averaged across epochs within participants with outliers (> 2 SD of the mean) rejected. This appears to improve the precision of the OMFs (not done in our previous paper, e.g.^8^). To obtain group averages, we then averaged across participants, to compute the event-related modulation of microsaccades with equal contribution from each participant.

#### Microsaccade reaction time (msRT)

Quantitative measures for the microsaccade inhibition duration were computed using a method introduced in^8,31^. Microsaccade RT (msRT) was calculated per epoch as the latency of the first microsaccade after stimulus onset in a window of 200–1,000 ms as the inhibition release interval. Epochs with no microsaccades in the specified window were not included in this calculation. In computing error bars for the RT values averaged across subjects, we applied the Cousineau method, which controls the between-subject variance and allows a better representation of within-subject effects^34^. In this method, data are first normalized by subtracting each subject’s mean RT and adding the group mean RT across all conditions and subjects. The standard error is calculated over the normalized data, and is multiplied by Morey’s correction factor^35^.

#### Oculomotor deviance

For each potential probe (1–8), we computed for each observer a measure of Oculomotor Deviance to the other potential probes. This deviance was based on the Oculomotor Modulation Functions (OMFs, see above) and primarily the microsaccade rate modulation. For individual subject data, pooled across the 4 experiments of 1-Reference (only the epochs with the reference face itself), 2-Face, 3-Name, and 4-City (Fig. 1a), the 3 Deviance measures computed over the OMFs were: (a) *Deviance(others)* = *mean squared difference from other probes’ average*, divided by the mean; (b) *Deviance(Reference)* = *the mean squared difference from the reference*, divided by the mean; (c) *Deviance(Combined)* = *Deviance(Others)—W*_*ref*_ × *Deviance(Ref)*, *W*_*ref*_ = 0.75, with deviance units referring to the units of the OMFs (e.g. saccade rates, saccade/s). The rationale for the combined deviance (c) is to maximize the difference from the average of the other potential probes and to minimize the difference to the Reference, which is assumed to reflect the individual pattern of oculomotor modulation (OMF) in response to a familiar image. The deviance measures described above were computed for each participant in the study (as shown in the results, Fig. 4) in a temporal window of 0–1,000 ms.

We developed another measure of *Relative Deviance* to provide a confidence measure for choosing the highest deviance as the familiar probe or deciding on “innocence” (none found). It is defined as the difference between the oculomotor deviance (as in Fig. 5) and the average deviance in the other competitors expressed in multiples of the standard deviation (SD) for the deviance across the other competitors (Fig. 6).

### Identification and classification analyses

We developed separate analyses for *identification* of the mock terror target for each participant in the study group, and for *classification* of “innocent” vs. “guilty” when all participants were taken together, i.e., to discriminate between the study and control participants. For identification, we determined the familiar mock terror target for each observer in the study group as the item (1–8) that shows the highest deviance (combined measure), when data were pooled across the three experiments (Face, Name, and City) and combined with the data from the reference experiment. When the correct probe was not the most deviant, we checked and reported if it is the second most deviant or the “runner up”. This yielded performance measures for the group, i.e. the percentage of observers with a correct identification, or when including the “runner ups”. Performance was classified using the relative deviance measure (combined) computed for each observer (a single number) and classification was done via a single criterion. We investigated the performance of this classification via the “area under the curve” (AUC) of the Receiver Operating Characteristics (ROC) function.

### Statistical assessment

For assessing the significance of the difference in the microsaccade rate modulation functions of familiar vs. the average of the unfamiliar faces, we used a nonparametric cluster-based randomization test^13,33,36^ as follows: For each time point, we calculated a paired t-test between the two rate functions. We then identified clusters of adjacent time points showing a significant t-value, and calculated the cluster-level statistics by summing all the t-values within a cluster. Then we randomly permuted (1,000 permutations) the labels of the data (i.e., depending on whether each value belonged to the familiar vs. the unfamiliar faces’ average), recalculated the cluster-level t-value, and generated a histogram of the test statistics across the permutations. We then computed the p value as the fraction of permutations in which the original cluster-level t-value was exceeded by that of the permuted data.

### Consent to publish images

Permission is granted by YSB and GR to publish the images in Fig. 1, including their faces under a CC by an open access license, and to publish the images in all formats, both print and digital. YSB and GR state that they appear in the facial photos of Fig. 1 and have the copyright for those pictures.

## Results

In the following, we report the results of (1) the general trends (group averages); (2) an individual analysis of familiarity based on oculomotor deviance for identification of the mock terror targets; (3) comparing the identification methods; (4) investigating the time course of identification; and (5) classification of “innocence” of the control vs. the study groups.

### The effect of familiarity on the oculomotor response

**Figure 2:**
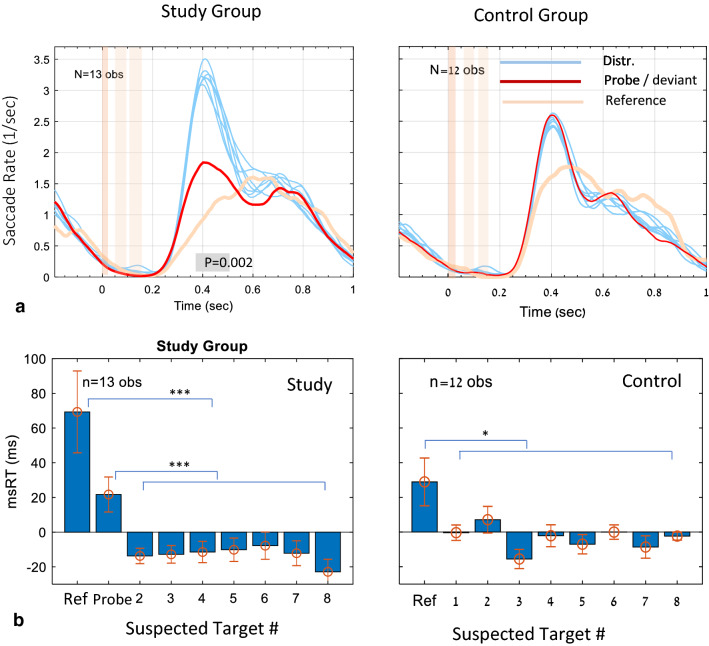
The effect of familiarity on the microsaccade rate modulation (**a**) and microsaccade RT (**b**), as group averages for the Study (left column) and Control (right column) groups. (**a**) Microsaccade rate modulation for the “mock terror” probe (in red), the universally familiar face (in beige) and the 7 “mock terror” distracters (light blue). For the Control group, the most deviant item for each observer was taken as the suspected probe (in red). The data for the probe and distracters were averaged across the 3 experiments (Face, Name, City) and across the 3 runs per observer and then averaged across observers. The “reference” was extracted from the universally familiar face responses in the reference experiment; Time zero represents stimulus onset, with shaded beige areas illustrating the stimulus image and mask times. The gray bar in (**a**) on the left panel indicates the significant cluster showing difference between the universally familiar face (in beige) and the average of all other faces (*p = 0.002, nonparametric permutation test, see “Methods” section), with similar but smaller effect (*p =  ~ 0.01, not shown) found for the mock-terror target; the similar comparison in the control group did not show significance. (**b**) Microsaccade RT (msRT) group averages, based on the latency of the first microsaccade in the range 200–1,000 ms post stimulus, for the data shown in (**a**), including the Reference, the probe (Study group only) and the 7 distracters. Data were averaged and normalized (demeaned) per observers, then averaged across observers, with error bars denoting 1SE across observers (see “Methods” section). Note in (**a**) the stronger and prolonged inhibition for the reference (in beige) for both groups and for the Probe (in red) for only the study group, as compared to the distracters (light blue). Note in (**b**) the significantly longer msRT for the Ref for both groups, and for the probe in the study group.

The group averages of the microsaccade effects of familiarity are shown in Fig. 2 (see the caption for details). For the study group, there were two familiar stimuli: the probe and the reference, whereas the control group was only familiar with the reference, and the item for comparison was selected as the most deviant item for each observer. The data for the reference for both groups were extracted from the universally familiar face responses in the separate reference experiment, whereas the data for the probes were pooled across the face, name, and city experiments.

The results of the microsaccade rate modulation are shown in Fig. 2a. As shown, there was a prolonged inhibition for the reference (in beige) in both groups, compared to the seven distracters (superimposed in light blue). The probe (in red) showed a prolonged inhibition for the study group but was indistinguishable from the distracters for the control group. These differences were statistically significant in the study group (Fig. 2a).

The results for the microsaccade RT are shown in Fig. 2b. The microsaccade RT (msRT) is the average across trials of the latency of the first microsaccade in the window of inhibition release (200–1,000 ms post stimulus) for the data shown in Fig. 2a. These msRT values were then averaged across observers within a group (see “Methods” section and caption). As shown, the reference was delayed in both groups compared to all the other items, excluding the probe in the study group, which showed a prolonged inhibition. The effects were significant, compared to the average across distracters, with p < 0.0005 for the reference and probe in the study group, and p = 0.015 for the reference in the control group (non-parametric permutation test, see “Methods” section).

### Identification of mock terror targets by the oculomotor deviance

The identification of the mock terror targets in the study group, using oculomotor measures extracted in passive viewing, was the primary goal of the study. The targets were chosen by each participant and were unknown at the initial analysis stage.

**Figure 3:**
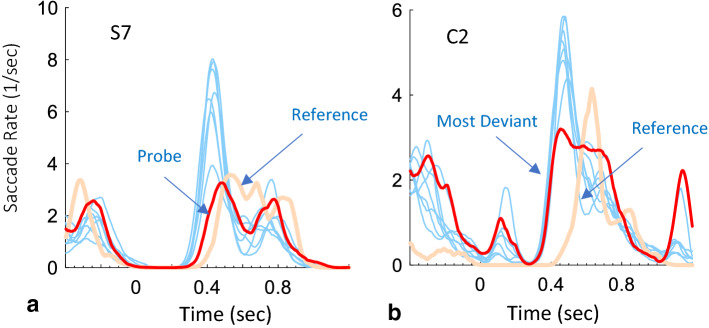
Individual rate modulation functions examples of (**a**) a study group subject S7 and (**b**) a control group subject C2, with data from the 3 experiments (face, name, residence) pooled together. The responses to the Probe/Deviant (red color) and the Reference (beige color) are marked via arrows. Note the similarity of the Probe and Reference for the study subject (in **a**) as compared to the distracters (light blue). Note the difference between the most deviant (in **b**) and the reference. This difference allows us to distinguish between deviance that stems from familiarity and other types of deviance, for the purpose of classifying the innocent.

**Figure 4:**
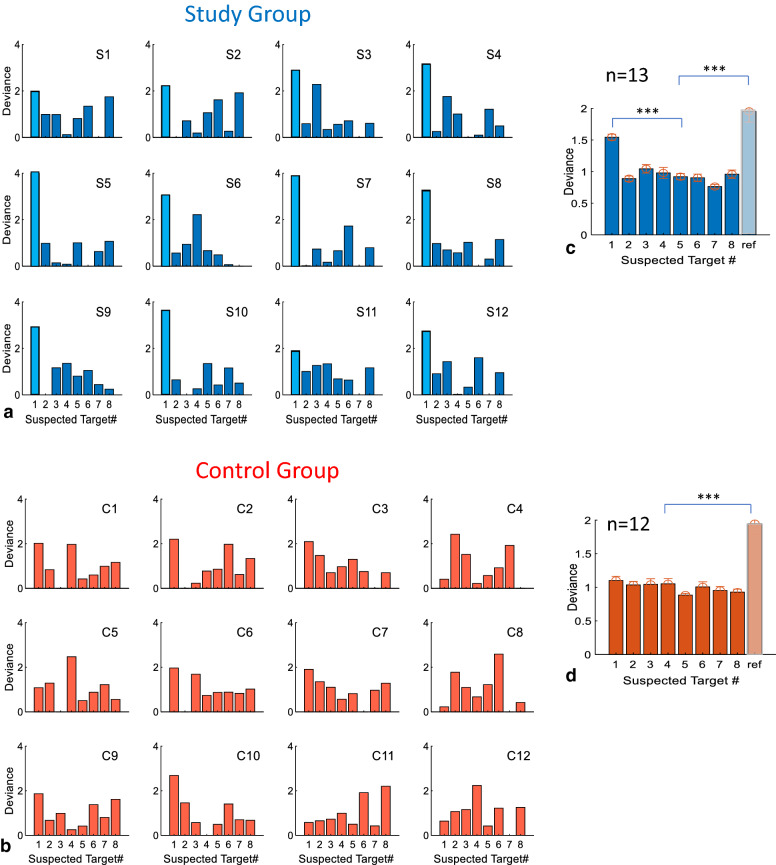
Identification of the mock-terror probes via oculomotor deviance. Individual oculomotor deviance plots are shown for the study group (**a**, blue) and the control group (**b**, orange). The deviance was calculated for each potential probe (1–8) as a weighted difference of its microsaccade rate modulation function deviance from the average of other potential probes, and from the reference, in rate (saccade/s) units, in the temporal window of 0–1,000 ms (see “Methods” section). In (**a**), the probe, i.e. the correct mock-terror target, which was randomly chosen, was moved to #1 and highlighted for clarity. Note that the #1 deviance is the highest for all subjects in the Study group, while there is no single most deviant number across all the controls. (**c,d**) group averages of the data in (**a**,**b**) respectively. The probe (#1) was found significantly higher than each of the other potential probes (p < 0.00005) in (**c**), but not in the control group (**d**). The reference deviance was significantly higher than all the other potential probes for both groups (**c,d**, p < 0.00005 for both).

At first, we inspected the individual OMFs (Oculomotor Modulation Functions, see “Methods” section) for microsaccades for the 3 probe types (face, name, and city) pooled together and for the reference, and we selected the most deviant potential probe that was also the most similar to the reference, without knowing the participant’s choice. An example of data from one study subject (S7) is shown in Fig. 3a, showing the microsaccade rate modulation for the irrelevant (in light blue), the probe (in red), and the reference (in beige). In practice, all stimuli were plotted in different colors except for the reference, since the probe was not known at the time of the initial manual analysis. As shown, the OMF of the probe stands out, or is the most deviant from the rest of the candidates (in light blue), and at the same time, it is the most similar to the reference (in beige). This manual process, based on visual inspection by the experimenter alone, yielded 100% correct identification, although a few cases were not totally clear and needed some guessing. We then developed an automated analysis that does a detection of deviance and similarity to the reference. This analysis, which identified the mock terror targets for each observer (Fig. 4), was based on the deviance in the Oculomotor Modulation Functions (OMFs), and primarily the microsaccade rate modulation functions averaged across epochs (see “Methods” section). For every observer, the deviance was calculated for each potential probe (1–8) as the combination (weighted difference) of its OMF deviance from the average OMFs of other potential probes, and from the reference (see “Methods” section). This measure is not based only on prolonged inhibition; it also examines the full shape of the OMFs: it determines how one OMF is the most different from the others and the most similar to the reference (used as a model of the individual responses to familiarity). In the results shown in Fig. 4, the correct item for each participant in the study group, i.e. the probe, was moved to #1 for clarity. As shown, this probe (light blue) was the most deviant in all cases, implying 100% success in identification. In comparison, there was no single most deviant item across the different control group observers.

### Comparing methods for identification of mock terror targets

**Figure 5:**
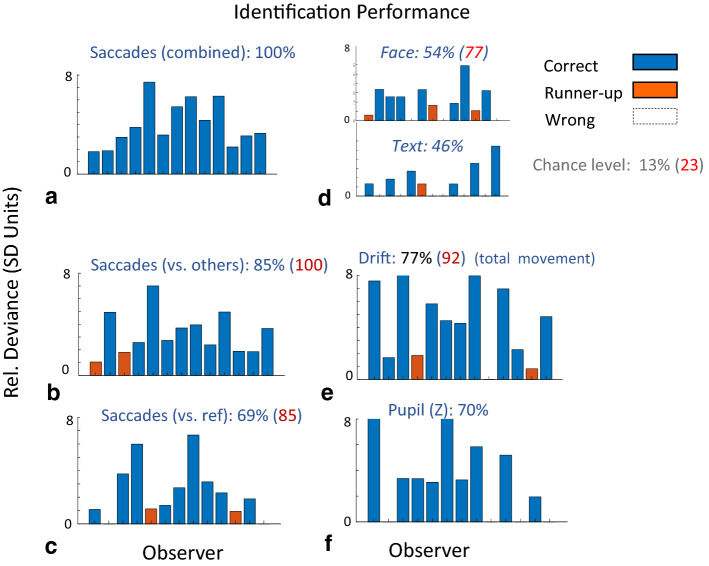
Comparing methods for identification of the probes. The bar plot for each method depicts the relative deviance of the “winner” (most deviant, in blue) or “runner-up” (second most deviant, in red), defined as the difference of its oculomotor deviance (as in Fig. 4) from the average deviance of the other competitors expressed in standard deviation units (SD of deviance across competitors). An empty bar is shown for identification error (i.e. when the correct identification is not the “winner” or “runner-up”). For all plots except d, the data from the 3 probes were pooled together. (**a**) The combined saccade deviance effect, yields 100% correct identification; (**b**) Deviance from others (see “Methods” section), yields 100% when 2 runnier-ups are included; (**c**) deviance from the universally familiar reference (see “Methods” section), yields 85% when 2 runnier-ups are included; (**d**) the isolated contribution of the Face and Text (name, city) to identification; (**e**) deviance of the total movement of the eyes (drift and saccades), yields 92% when 2 runnier-ups are included; (**f**) pupil size relative deviance, yields 70% correct identification. Note that the probability for correct identification of the probe for all N = 13 subjects is 1/8^N^ (< 10^–10^), which is equivalent to 13% chance-level identification (~ 1.63 observers on average).

To assess the confidence of the deviance measures, e.g., as a marker of the concealed familiar item, or for the lack of “innocence”, we need to take into account not only the highest deviance (Fig. 4), but also the “competitors”, i.e., the potential targets (probes) with high but not the highest deviance. This is because choosing the highest deviance could very likely be a mistake if a “runner up” (the second highest) with a high deviance exists. We therefore developed another measure of Relative Deviance (see “Methods” section) to provide a confidence measure in choosing the highest deviance as the familiar item, or in deciding on “innocence” (none found). We then used this measure to assess and compare different identification methods in the mock terror experiment based on the Relative Deviance for different OMFs. The results, shown in Fig. 5, consider also “runner ups” (in red, see “Methods” section), and report the accuracy measures of the percentage of observers making a correct identification. The highest accuracy rate (100%) was obtained with the *Deviance(Combined)* measure, which takes into account both the deviance from the others and the similarity to the OMF of the reference (Fig. 5a). We found a reduced identification performance when looking only at the deviance from the reference (69%, Fig. 5c), or from the “others” (85%, Fig. 5b), or when analyzing the data for the Faces (54%), and Text (46%), separately (Fig. 5d). To examine the sensitivity of the method to microsaccade detection, we also assessed the Drift (total movement, including microsaccades, see “Methods” section), which yielded a somewhat reduced performance (77%, 92 with runner ups, Fig. 5e). Finally, a similar analysis, using the pupil size modulation, yielded 70% identification (Fig. 5f), suggesting that the pupil size also contains information on familiarity in our setup. In comparison to all these results, the chance level for the percentage of observers making a correct identification is 13%, and 23% if “runner ups” are included; identifying the correct mock terror target in 2 observers (15%) will already correspond to p < 0.05.

### The time course of mock terror target identification

**Figure 6:**
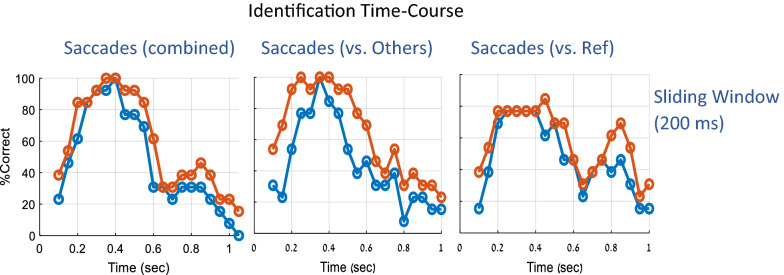
The time-course within a trial of probe identification. The percentage of study group participants with correct identification (blue) or correct identification when the runner-up is included (orange) are plotted as a function of time of deviance estimation computed in a sliding window of 200 ms, in 50 ms steps. This analysis was applied in 3 deviance calculation methods (as in Fig. 5a–c): saccade, combined effect, saccades vs. others, and saccades vs. reference. Note that the sliding window data indicate that the deviance information for identification is maximized around 400 ms, which is the approximate time of the oculomotor inhibition release. Note also that above chance performance (for runner-ups, where chance is 23%) could be observed during inhibition onset (below 200 ms).

Based on the deviance measure, we conducted an additional analysis to investigate the time course within the trial of the identification process: to what extent it depends on a specific time window and at what processing stage the familiarity is identified and expressed in the OMF. The results are shown in Fig. 6 and explained in its caption. The analysis was done by computing the deviance in a sliding window (200 ms, 50 ms steps), shown in Fig. 6. As shown, the sliding window data indicate a peak in identification performance around 400 ms post stimulus, with information available starting from 150 ms. Note also that above chance performance could be observed during inhibition onset (below 200 ms).

### Classification of “innocence” by oculomotor deviance

**Figure 7:**
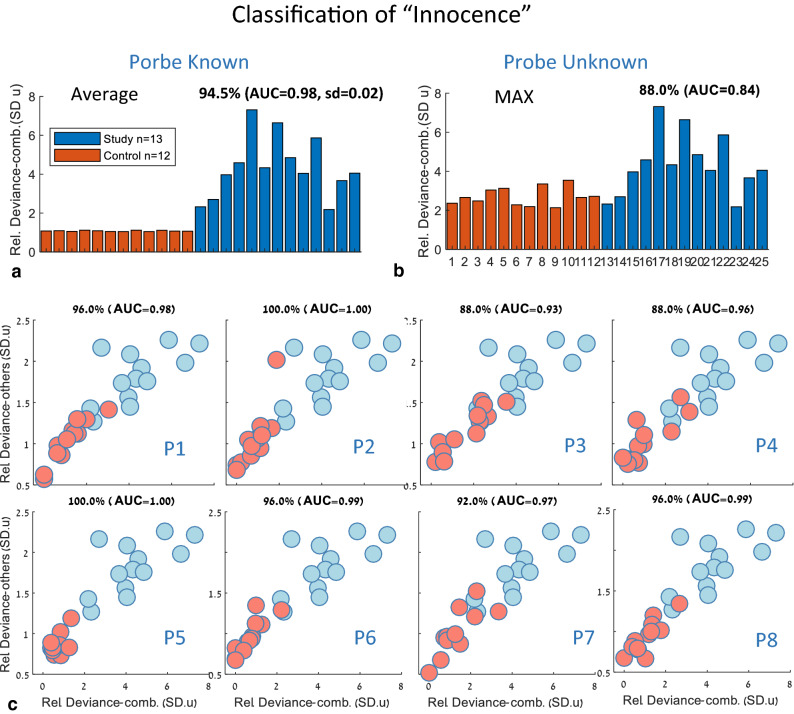
Classification of “innocence” of the control vs study groups. Two scenarios were considered: (1) There is only one known terror target (“guilty knowledge”), and “innocence” is defined in relation to it; (2) Target unknown, and all the associated probes are possible (rare scenario). (**a**) Classification for the single terror target scenario. The relative deviance (in SD units, combined measure as in Fig. 5), is plotted for all controls (as average across all 8 possible probes), and study subjects (for the correct probe). The quality of this classification is given by the average AUC (see “Methods” section) of 0.98 ± 0.02. This average was derived from the data in (**c**). (**b**) Classification based on the MAXIMUM deviance, assuming the terror target is unknown (the rare scenario, (2) above). The maximum relative deviance was selected for each control subject, while the terror target for the study subjects was known (and therefore identical to (**a**)). This yielded 88% classification and AUC = 0.84. (**c**) Scatter plots of deviance data for all 8 possible probes for the controls (orange) and the known terror target for the study subjects (light blue, same data in all plots), for scenario (1) above. The X-axis shows the relative Deviance-combined effect and the Y-axis, the Deviance from the others (see “Methods” section). The percent correct and AUC values based on the combined effect appear in the title for each plot.

In addition to identification of the mock terror target individually selected by each participant in the study group, we had a control group that was “innocent” and only participated in the same eye tracking experiments as the study group. Our goal was to classify the group of each participant, i.e. determine whether the participant belonged to the “terrorist” study group or to the “innocent” control group. An example illustrating the essence of this process appears in Fig. 3, showing data from one “terrorist” (S7) and one “innocent” (C2) subject (Fig. 3a,b). As shown, S7 had one clear deviant (in red), which was very similar to the reference (in beige), whereas C2 had a somewhat deviant item (in red), which was very different from the reference. These differences allow for a clear distinction between the two. The results for all subjects are shown in Fig. 7. We considered two scenarios: (1) There is one specific known terror target (“guilty knowledge”), and “innocence” is defined according to the familiarity with it (common scenario); (2) The terror target is unknown, and “innocence” is defined according to the familiarity with any of the potential probes (rare scenario). We used the Relative Deviance measure of *Saccades(combined)* calculated for each observer (see “Methods” section and Fig. 5) to classify innocence via a threshold criterion. In addition, we conducted ROC analysis to obtain the “Area Under the Curve” (AUC), which is a popular tool for assessing classification performance^1^. For the “target known” (guilty knowledge) scenario, we averaged across all possible probes (1–8) and obtained an average AUC of 0.98, which is considered high (Fig. 7a). This average was calculated across AUC values calculated separately for each potential probe (P1–P8, Fig. 7c). For the rarer scenario of “target unknown” (Fig. 7b), we used the potential probe with the maximum deviance for each participant as the selected choice. The results show 88% correct classification and a lower AUC of 0.84.

## Discussion

In a mock terror experiment involving 25 participants, of whom 13 were pre-exposed to three "terror target" probes, we demonstrated the successful application of an Oculomotor Inhibition-based paradigm to CIT as a “proof of concept”, with performance comparable to the current best CIT methods. In the following, we discuss different issues raised by the results, in comparison to other CIT methods and the limitations of the study.

### Oculomotor inhibition and the deviance-based concealed information test

The concealed information test is a method used to reveal memory traces based on a deviant physiological or behavioral response to familiar or personally significant probes. Although several physiological measures were applied as well as some novel eye tracking techniques^25^, measures based on fixational eye movements and specifically on the phenomenon of OMI were never applied to CIT. Here we demonstrate, for the first time, OMI-based CIT in a realistic mock terror experiment. Our initial approach was to follow our previous study in which we showed prolonged microsaccade inhibition as well as an earlier onset of inhibition for familiar faces^2^. To obtain a direct measure of the release from inhibition latency, we averaged the onset times of the first microsaccade in the release period (200–1,000 ms following stimulus onset) of each epoch of stimulus presentation (msRT). The results of this analysis revealed significant group average effects (Fig. 2b) but were not accurate at the individual level as needed for practical CIT. Instead, we introduced a new measure of “Oculomotor Deviance”, which is not specific to the discrete microsaccade events, and it works for any continuous Oculomotor Modulation Function (OMF), including, for example, the modulation in pupil size (Fig. 5f) or the total movement (drift) of the eyes (Fig. 5e). This measure is the deviance of the OMF of one item from the average of the rest, quantifying how one item stands out as an oddball compared to the others, regardless of the OMF shape. This novel approach produced the results of a 100% success rate in identifying the probes in the study group. Its advantage over the microsaccade RT measure stems from the individual differences in the shape of the OMF, which does not always conform to the standard pattern of inhibition and release in standard time windows. Another advantage of the deviance analysis is in the natural way in which it can integrate the typical response to a familiar pattern as a reference. This issue is discussed in a separate section.

### Oculomotor deviance and the orienting response

The physiological measures that are currently used in CIT methods are based on physiological deviance, which is often attributed to an orienting response, assuming that the familiar is an oddball among non-familiar distracters and has personal significance, which attracts attention (e.g.^37,38^). It is not yet unclear whether the OMI or its deviance reflects an orienting response similar, for example, to the P300-based CIT^23^. In one study, the magnitudes of the P300 and the OMI strength in a visual oddball task were found to be weakly related^39^. Our results with different types of visual, auditory, and cross-modal oddball stimuli revealed a prolonged OMI as a function of the perceptual deviance^40,41^, but this marker of deviance could also be related to the pre-attentive Mismatch Negativity (MMN) potential of around 200 ms, observed even when attention is distracted from the oddball stimuli, or in patients with minimal consciousness^42^, i.e. without orienting. There are some indications that the OMI response to familiarity could be early, as in the early measures of the onset of inhibition (< 200 ms) in our previous study for the universally familiar faces^2^. However, this effect was small, and we did not find a similar effect in the current study for the response to the mock terror probes in the study group. Nevertheless, the analysis of the time course of identification (Fig. 6) shows that a significant identification occurs already around 200 ms (with a 200 ms integration window). More research is needed to clarify the relation of the OMI to orienting, oddball response, and perhaps even a sub-conscious response to familiarity.

### Individual oculomotor response to familiarity as a fine-tuning mechanism

Despite the general finding that familiarity prolongs the OMI, for both the universally familiar face^2^ and the mock terror probes (Fig. 2a), we found significant individual differences in the oculomotor response to familiarity in our data (see the example in Fig. 3, see also^43^). Using these individual oculomotor functions as a reference for comparison could be useful for two reasons. First, it will counteract and reduce the effect of deviance due to arbitrary noise. Second, it will counteract deviant responses due to random saliencies in the stimulus that do not necessarily conform to the oculomotor response to face familiarity. This could happen, for example, when the saliency of the stimulus stems from low-level processes such as contrast (even at a local location in the image), in which case the OMI is typically shortened rather than prolonged^8^. This implies, for example, that a strongly deviant response that is vastly different from the reference might not be related to a familiar probe. We therefore measured the oculomotor response of each observer to a universally familiar face in a separate experiment and used it as an individual reference. To identify the probe, we then combined the oculomotor deviance (one item against the average of the others) with the similarity to the reference, to maximize both (*Deviance(combined*), see “Methods” section). This resulted in improving identification from 85 to 100% (compare Fig. 4a,b). It is still unclear whether this method could be effective for rejecting perceptual deviance not related to familiarity, which nevertheless elicits an orienting response, e.g. due to some personal interest or individual differences in visual salience^44^. This issue requires further investigation.

### Essential components and comparison to other CIT methods

There are several components of our CIT method that are essential for its high performance, some of which make it different from other CIT methods.

1. *Passive viewing* Our method does not use any task and the observers are only asked to fixate and pay attention to the stimuli. As far as we know, this is the first CIT method that is totally passive, i.e. without a task. In comparison, the P300 CIT methods typically use stimuli (targets and pop quizzes) to which the participant must respond by pressing a button, and when the targets are omitted (but the pop quizzes are left), the CIT performance degrades in some cases^45^. Although our subjects are only asked to fixate and pay attention, the method could be described as “passive-attentive”^42^ because attention is not directed away from the stimuli. Importantly, since no question is asked and stimuli are presented on the fringe of awareness, the purpose of the test could be hidden from the subject. However, in the future we might need to add active “catch trials” to detect countermeasures (see the “Limitations” section).
2. *Short testing* The test is short, about 25 min in total, including the reference and the 3 probes, and stimuli are presented at 1 Hz, i.e. 1 s per trial, which is much shorter than a typical P300 CIT, which takes > 2 s per trial with a response^23^.
3. *Remote tracking* The tracking was applied remotely, without attaching any wires or devices, which is the advantage of using any video-based eye-tracking CIT. It should be noted that the current study used the most accurate eye tracking conditions available, including 500 Hz tracking (Eyelink). Thehead was stabilized by a chinrest, to detect the smallest microsaccades and to ensure accurate rate modulations. However, we believe that head stabilization will not be critical for accurate tracking in the future.
4. *Multiple and infrequent probes* We used 3 probes (face, name, and city; see Methods) to obtain a significantly higher identification performance than from a single probe. We obtained 54% of the correct subjects for the face alone, and 46% for the text items (Fig. 5d; the chance level is 13%), compared to 100% for all combined. The probes were presented infrequently, one in eight like in a lineup, which is rarer than the typical P300 CIT of 1 in 6^23^ and was made possible due to the high speed of presentation (1 Hz without pauses).
5. *Fringe of awareness presentation* This is similar to previous studies of Bowman et al.^18,46,47^. We presented all stimuli on the fringe of awareness using backward masking with a very short (10 ms) stimulus-of-interest presentation, followed (60 ms gap) by two successive masks (Fig. 1a). This resulted in reduced visibility, which observers rated in our previous study (identical paradigm, for the faces part) as “barely visible”, on average^2^. The use of masking followed a previous attempt to detect face familiarity without masking, which did not show good results^2^, and another partly unsuccessful attempt to use RSVP (as in^18^, but using OMI and not P3 ERP). Bowman et al.^46^ have recently shown that famous (familiar) faces, but not novel (unfamiliar) faces break into awareness in RSVP, resulting in large differences between brain responses to the familiar and unfamiliar. We believe that the same principle works in our case, with the familiar stimuli (the reference, as well as the probes) but not the unfamiliar tending to break into awareness through the masks, resulting in an amplified difference and a sensitive method to dissociate between them.
6. *Accuracy* Our results showed 100% correct identification of the mock terror target in the study group (n = 13), i.e. the correct mock terror target number detected for all study participants (and hence no false alarms or misses, Fig. 5a). This result strongly depends on the prior knowledge of having exactly one correct mock terror target for each subject, and knowledge of the association between probes, i.e. the face that goes with the person’s name and the city’s name. Similar results of perfect identification were previously obtained using P300 ERP^48^, although with some differences in paradigm, and especially the 1-of-4 probe rate vs 1-of-8 in our study. More challenging in our study was the classification of “guilty” and “innocent” among the 25 participants, which we analyzed under two different scenarios. In one scenario, which is very realistic in crime investigations, the probes are known and our result of AUC = 0.98, on average, across the 8 possible probes (Fig. 7a) is in the highest range of previous studies including P300 CIT (see^1^ for a meta-analysis). When the probes are not known, i.e. when distinguishing between “innocent” people not familiar with any of the stimuli and those who are, our performance was lower, 88% with AUC = 0.84 (Fig. 7b), similar to previous studies (e.g. 83% correct, AUC = 0,87 in a mock terror experiment^48^). Overall, the accuracy we obtained was in the high range of previous studies^1^.
7. *Robustness* Part of our data analysis was developed post-hoc, i.e. to produce optimal results in identification and classification for the current sample. Nevertheless, the core method is quite robust, and the extensive analyses presented are intended to convince others that it was not tuned tightly to the specific sample. First, the microsaccade rate modulation functions were identical to those we used in previous studies^8,13^, except for removing outliers (above 2 standard deviations). Second, we applied a novel measure of “relative deviance” (see “Methods” section), which has no free parameters. There was only one free parameter: the relative weight assigned to the deviance from other candidates compared to the weight *W*_*ref*_assigned to the reference (see “Methods” section. When this parameter was set to *W*_*ref*_ = 1 (equal weight) instead of *W*_*ref*_ = 0.75, the identification was missed by one observer (but it was still 100% when considering the runner up) and the classification performance was reduced only for the “target unknown” scenario (to 84%, AUC = 0.74). For a weight of *W*_*ref*_ = 0.5, the identification remained 100% and the classification showed almost no change. When only the deviance measures were used without the reference (*W*_*ref*_ = 0), the identification was reduced to 85% (Fig. 5b) and the classification for “target unknown” was reduced to 80%, AUC = 0.8. Additional evidence for the robustness of the method can be derived from Fig. 6, showing how identification builds up over time, and from Fig. 5e,f, showing that quite good (but not optimal) identification can be derived from the total movement (microsaccade and drift), which is independent of the microsaccade detection method (Fig. 5e) and from the pupil size modulation (Fig. 5f).

### Limitations

This study could be regarded as a “proof of concept” for the use of the Oculomotor Inhibition (OMI) effect during fixation for detecting concealed information in a CIT. As such, it does have some limitations that need to be emphasized. The two main limitations are the lack of countermeasures or a deception experiment, and the post-hoc development of the data analysis algorithm.

#### The lack of a deception experiment

We did not test for deception in the current study; it is left for future work; however, we can nevertheless consider the possibility of different deception types, which can be divided into two categories: oculomotor and cognitive. Regarding “oculomotor deception”, we noted that although the Oculomotor Inhibition effect (OMI) for microsaccades during fixation is typically considered involuntary (e.g.^49^), there is evidence that the microsaccades themselves could be generated intentionally even to memorized locations^50^. In general, there could be different oculomotor ways to disrupt the method, e.g., by extensive blinking, moving the eyes intentionally with saccades or microsaccades from side to side, and fixating on one peripheral point; however, such oculomotor disruptions should be possible to detect. As for cognitive strategies, we believe that with our method it is difficult to deceive, similar to a previous study that presented stimuli on the fringe of awareness and largely prevented deception^18^. The reason is that willfully modulating the neurophysiological or the involuntary oculomotor response to specific stimuli requires conscious control; this is possible to obtain perhaps with a slow presentation, but it appears almost impossible with stimuli presented on the fringe of awareness. More research is needed to determine whether this method is resistant to cognitive countermeasures, which would be harder to detect, and whether such measures, if they exist, could be overcome by adding a demanding task that will make deception difficult and detectable.

#### The post-hoc data analysis

One potential weakness of the study is the post-hoc development of the data analysis procedure, as described in the Methods section. One may be concerned that the methods were optimized to the specific sample and produced “overfitting”, e.g., 100% identification, which will not generalize to another sample. How significant is this weakness? This depends on the number of “free parameters” and choices made in the analysis. We analyzed and discussed in detail the effect of the relevant parameters on the results, i.e., the robustness of the method. Following this analysis, we concluded that although some overfitting of the methods could be responsible for the 100% correct identification and for the high AUC values in classification, highly significant results could be obtained effortlessly and in different ways, e.g., with the ‘drift’ (total movement) of the eyes (Fig. 5e), or even with pupil size modulation (Fig. 5f); see the “Robustness” section above. One should also note that the oculomotor deviance whose measure was later developed (post-hoc) was the method that we first used perceptually to determine the correct mock terror target. Each of the authors reached a 100% independent identification by manual visual inspection without knowing the correct results (which were “concealed”, see the “manual analysis” under the “Results” section). This also strengthens our confidence in the results.

Another limitation is the relatively small sample of 25 subjects divided into 2 groups, 12 and 13. This sample is not uncommon in oculomotor studies (e.g.^8,33^), it is smaller than many CIT studies (e.g.^45^), but it is similar to others when considering the number of participants per condition (e.g.^47^). It should be noted that the results on identification are not based on group averages, but instead on statistically significant identification of the mock terror target for each participant in isolation. Nevertheless, the small sample is a limitation for the classification of “innocence”.

A fourth limitation concerns the intensive “learning phase” of the study participants, which could make our results different from those of CIT in real life due to their possible dependence on priming. Since in our paradigm the “terrorists” learned the probes just minutes before being tested (~ 20 min of learning, followed by ~ 25 min of CIT), the learning via text and video exposure could have produced a strong priming effect that elevated performance, compared with real-life CIT (see^51^ for face priming that lasts minutes and days). We think that this is unlikely, at least as a major factor, since we got similar results with the universally familiar face that we used for reference (see examples in Fig. 3), suggesting that the familiarity induced by recent learning was similar to the familiarity induced by an old exposure. Moreover, in our results the reference appeared to induce a longer OMI than with the probes (Fig. 1b), suggesting that priming (or familiarity via a very recent exposure) is not more effective than long-term familiarity. On the contrary, it is possible that our method will be more effective with stimuli of personal significance, which is the typical case in real-life CIT. This could be investigated in future studies. Another possibility is that the study participants might have understood the connection between the two parts of the experiment and might have been highly motivated to “comply” and focus their attention, thus differentiating them from the control group to produce a good classification. This possibility, however, is inconsistent with the similar deviance found for the reference between groups (Fig. 4c,d), but it should be investigated in the future.

## Conclusion and future directions

Our results show, for the first time, an oculomotor-inhibition (OMI)-based CIT with high accuracy. The novel method presents stimuli on the fringe of awareness in passive viewing, it uses remote eye tracking, and utilizes an individual response to familiarity as a reference. As a “proof of concept” it has some limitations. More research is needed to determine if our method is resistant to cognitive countermeasures and is effective in ignoring arbitrary perceptual deviance not related to familiarity. In general, it will be necessary to clarify the relations between OMI and orienting, the oddball response, and perhaps even a subconscious response to familiarity, in order to develop a future real-life CIT method based on involuntary eye movements—one that works in a totally stealth manner.

## Data Availability

The stimuli are public and can be obtained upon request. The data are presented graphically in the manuscript in detail, including all the individual results; numerical representations of these graphs can be obtained upon request as applicable.

## References

[1] Meijer EH, Selle NK, Elber L, Ben-Shakhar G (2014). Memory detection with the concealed information test: A meta analysis of skin conductance, respiration, heart rate, and P300 data. Psychophysiology.

[2] Rosenzweig G, Bonneh YS (2019). Familiarity revealed by involuntary eye movements on the fringe of awareness. Sci. Rep..

[3] Barlow HB (1952). Eye movements during fixation. J. Physiol..

[4] Steinman RM, Haddad GM, Skavenski AA, Wyman D (1973). Miniature eye movement. Science.

[5] Rolfs M (2009). Microsaccades: Small steps on a long way. Vis. Res..

[6] Hafed ZM, Clark JJ (2002). Microsaccades as an overt measure of covert attention shifts. Vis. Res..

[7] Pastukhov A, Braun J (2010). Rare but precious: Microsaccades are highly informative about attentional allocation. Vis. Res..

[8] Bonneh YS, Adini Y, Polat U (2015). Contrast sensitivity revealed by microsaccades. J. Vis..

[9] Yuval-Greenberg S, Merriam EP, Heeger DJ (2014). Spontaneous microsaccades reflect shifts in covert attention. J. Neurosci..

[10] Betta E, Turatto M (2006). Are you ready? I can tell by looking at your microsaccades. NeuroReport.

[11] Valsecchi M, Betta E, Turatto M (2007). Visual oddballs induce prolonged microsaccadic inhibition. Exp. Brain Res..

[12] Valsecchi M, Turatto M (2009). Microsaccadic responses in a bimodal oddball task. Psychol. Res..

[13] Yablonski M, Polat U, Bonneh YS, Ben-Shachar M (2017). Microsaccades are sensitive to word structure: A novel approach to study language processing. Sci. Rep..

[14] Bonneh Y, Adini Y, Fried M, Arieli A (2011). An oculomotor trace of cognitive engagement. J. Vis..

[15] Rolfs M, Kliegl R, Engbert R (2008). Toward a model of microsaccade generation: The case of microsaccade inhibition. J. Vis..

[16] Engbert R, Kliegl R (2003). Microsaccades uncover the orientation of covert attention. Vis. Res..

[17] Valsecchi M, Turatto M (2007). Microsaccadic response to visual events that are invisible to the superior colliculus. Behav. Neurosci..

[18] Bowman H (2013). Subliminal salience search illustrated: EEG identity and deception detection on the fringe of awareness. PLoS ONE.

[19] Ben-Shakhar G, Elaad E (2003). The validity of psychophysiological detection of information with the guilty knowledge test: A meta-analytic review. J. Appl. Psychol..

[20] Klein Selle N, Agari N, Ben-Shakhar G (2019). Hide or seek? Physiological responses reflect both the decision and the attempt to conceal information. Psychol. Sci..

[21] Ben-Shakhar G (1977). A further study of the dichotomization theory in. Psychophysiology.

[22] Klein Selle N, Verschuere B, Kindt M, Meijer E, Ben-Shakhar G (2017). Unraveling the roles of orienting and inhibition in the concealed information test. Psychophysiology.

[23] Rosenfeld JP (2019). P300 in detecting concealed information and deception: A review. Psychophysiology.

[24] Nahari T, Lancry-Dayan O, Ben-Shakhar G, Pertzov Y (2019). Detecting concealed familiarity using eye movements: The role of task demands. Cogn. Res. Princ. Implic..

[25] Lancry-Dayan OC, Nahari T, Ben-Shakhar G, Pertzov Y (2018). Do you know him? Gaze dynamics toward familiar faces on a concealed information test. J. Appl. Res. Mem. Cogn..

[26] Seymour TL, Baker CA, Gaunt JT (2013). Combining blink, pupil, and response time measures in a concealed knowledge test. Front. Psychol..

[27] Cook AE (2012). Lyin’ eyes: Ocular-motor measures of reading reveal deception. J. Exp. Psychol. Appl..

[28] Artzi NS, Shriki O (2018). An analysis of the accuracy of the P300 BCI. Brain-Comput. Interfaces.

[29] Lukács G (2016). The first independent study on the complex trial protocol version of the P300-based concealed information test: Corroboration of previous findings and highlights on vulnerabilities. Int. J. Psychophysiol. Off. J. Int. Organ. Psychophysiol..

[30] Visconti Di Oleggio Castello M, Halchenko YO, Guntupalli JS, Gors JD, Gobbini MI (2017). The neural representation of personally familiar and unfamiliar faces in the distributed system for face perception. Sci. Rep..

[31] Bonneh YS, Adini Y, Polat U (2016). Contrast sensitivity revealed by spontaneous eyeblinks: Evidence for a common mechanism of oculomotor inhibition. J. Vis..

[32] Bonneh Y (2010). Motion-induced blindness and microsaccades: Cause and effect. J. Vis..

[33] Widmann A, Engbert R, Schroger E (2014). Microsaccadic responses indicate fast categorization of sounds: A novel approach to study auditory cognition. J. Neurosci..

[34] Cousineau D (2005). Confidence intervals in within-subject designs: A simpler solution to Loftus and Masson’s method. Tutor. Quant. Methods Psychol..

[35] Morey RD (2008). Confidence intervals from normalized data: A correction to Cousineau (2005). Tutor. Quant. Methods Psychol..

[36] Maris E, Oostenveld R (2007). Nonparametric statistical testing of EEG- and MEG-data. J. Neurosci. Methods.

[37] Lykken DT (1974). Psychology and the lie detector industry. Am. Psychol..

[38] Shakhar GB, Lieblich I, Kugelmass S (1970). Guilty knowledge technique: Application of signal detection measures. J. Appl. Psychol..

[39] Valsecchi M, Dimigen O, Kliegl R, Sommer W, Turatto M (2009). Microsaccadic inhibition and P300 enhancement in a visual oddball task. Psychophysiology.

[40] Kadosh O, Bonneh YS (2020). Involuntary markers of auditory surprise revealed by oculomotor inhibition. Sci. Rep..

[41] Bonneh Y (2013). Microsaccade latency uncovers stimulus predictability: Faster and longer inhibition for unpredicted stimuli. J. Vis..

[42] Bekinschtein TA (2009). Neural signature of the conscious processing of auditory regularities. Proc. Natl. Acad. Sci..

[43] Stacchi L, Ramon M, Lao J, Caldara R (2019). Neural representations of faces are tuned to eye movements. J. Neurosci..

[44] De Haas B, Iakovidis AL, Schwarzkopf DS, Gegenfurtner KR (2019). Individual differences in visual salience vary along semantic dimensions. Proc. Natl. Acad. Sci. U.S.A..

[45] Davydova E, Rosenfeld JP, Labkovsky E (2020). Necessity of the target discrimination in the P300-based complex trial protocol test for concealed information. Psychophysiology.

[46] Alsufyani A (2018). Breakthrough percepts of famous faces. Psychophysiology.

[47] Bowman H, Filetti M, Alsufyani A, Janssen D, Su L (2014). Countering countermeasures: Detecting identity lies by detecting conscious breakthrough. PLoS ONE.

[48] Meixner JB, Rosenfeld JP (2011). A mock terrorism application of the P300-based concealed information test. Psychophysiology.

[49] White AL, Rolfs M (2016). Oculomotor inhibition covaries with conscious detection. J. Neurophysiol..

[50] Willeke KF (2019). Memory-guided microsaccades. Nat. Commun..

[51] Mueller R, Utz S, Carbon CC, Strobach T (2020). Face adaptation and face priming as tools for getting insights into the quality of face space. Front. Psychol..

